# Breed differences in humoral and cellular responses of lambs to experimental infection with the gastrointestinal nematode *Teladorsagia circumcincta*

**DOI:** 10.1186/s13567-014-0137-0

**Published:** 2015-02-17

**Authors:** Albin Mostaque Ahmed, Simone Rocco Sebastiano, Torres Sweeney, James Patrick Hanrahan, Assumpta Glynn, Orla Mary Keane, Anindya Mukhopadhya, Kevin Thornton, Barbara Good

**Affiliations:** Veterinary Sciences Centre, University College Dublin, Belfield, Dublin 4 Ireland; Animal & Grassland Research and Innovation Centre, Teagasc, Athenry, Co. Galway Ireland; AGRIC, Teagasc, Grange, Dusany, Co. Meath. Ireland

## Abstract

While Texel lambs have increased resistance to infection with the gastrointestinal nematode *Teladorsagia circumcincta* compared to Suffolk lambs, the underlying resistance mechanisms are still unknown. The aim of this study was to compare parasitological, humoral and cellular responses of Texel and Suffolk lambs over time following a single experimental infection with *T. circumcincta*. Gastrointestinal nematode free (but not naïve) lambs received a single oral dose of 3 × 10^4^ infective *T. circumcincta* larvae. The variables examined included worm burden, mucosal and serum IgA, abomasal mast cells and eosinophils, haematological parameters and plasma pepsinogen. Texel lambs had significantly lower worm burden on day 14 and lower plasma pepsinogen concentration from day 14 onwards than Suffolks and their response in mucosal IgA to infection occurred earlier. The results from the study suggest that an earlier local IgA response in the Texel contributes to the resistant characteristics of the breed, while the increased level of plasma pepsinogen in the Suffolk lambs implies greater abomasal tissue damage arising from the nematode infection.

## Introduction

*Teladorsagia circumcincta* is among the most important gastrointestinal nematode (GIN) species affecting sheep production in temperate regions [1,2]. GIN infection has a negative effect on reproductive performance, milk production, body weight, carcass quality and survival [3,4]. Extensive use of anthelmintics as a control strategy has resulted in the evolution of anthelmintic resistance in various nematode species [5]. This together with consumer concern over drug residues in animal products has promoted interest in the development of alternative methods of GIN control, such as genetic selection for increased host resistance to GIN. A number of studies have already identified established breeds that are relatively resistant to various GIN species [6-9]. In Ireland, the Texel breed is more resistant to GIN infection than the Suffolk [6]. Identification of physiological markers associated with resistance would facilitate the classification of resistance status of individuals and thus contribute to the development of rapid reliable markers for use in national sheep breeding programmes. Resistance to nematode infection can be manifest as a combination of impaired larval establishment, inhibition of larval development, reduced worm fecundity and/or accelerated worm expulsion [10,11]. Resistant animals may have more efficient mechanisms for affecting some or all of these physiological processes. While the only direct method of identification of resistant animals is to measure worm burden, this is not practical for use in breeding programmes, as it requires animals to be sacrificed [12]. Faecal egg count (FEC) is positively correlated with worm burden [13] and has been proposed and used as a marker of resistance to GIN [14,15]. However, there are various limitations to FEC data including variability due to host factors (age, gender, immune status and stress), parasite specific factors (variability in species composition, fecundity, developmental stage), environmental factors (nutrition, climate), sampling accuracy and precision [16-21].

It is hypothesised that physiological processes that determine host control of worm burden will vary between resistant and susceptible animals following GIN challenge. Previous data have suggested that resistance to GIN infection depends on the activation of an effective Th2 immune response which elicits a humoral immune response and results in the recruitment of eosinophils and mast cells to the gastrointestinal mucosa and the local production of IgA and IgE antibodies [22]. Pepsinogen concentration in blood plasma reflects the extent of abomasal tissue damage [23] and is elevated in Suffolk lambs naturally infected with GIN in comparison to Texel lambs [24].

The objective of this study was to identify physiological markers in blood or abomasal mucosa that differ between Suffolk and Texel lambs following artificial challenge with infective *T. circumcincta* larvae that may be indicators of resistance/susceptibility to *T. circumcincta* infection.

## Materials and methods

### Ethical approval

All procedures described in this study were conducted under experimental license from the Irish Department of Health in accordance with the Cruelty to Animals Act 1876 and the European Communities (Amendments of the Cruelty to Animals Act 1976) Regulations, 2002 and 2005.

### Animals

All lambs (32 Texel and 29 Suffolk) were sourced from the flock of purebred Suffolk and Texel sheep maintained at Athenry Research Centre [6]. Lambs of both the breeds were born indoors from a synchronised mating programme and then all the lambs were moved to the same pasture for a 6-week period. Lambs were weaned at about 6 weeks of age and moved indoors where they were maintained on a concentrate-based diet with free access to water for the remainder of the experiment. Upon housing, faecal sampling *per rectum* was attempted on all lambs, but sufficient material was obtained from only 36 individuals (16 Texel and 20 Suffolk). All lambs were then treated with Ivermectin (Oramec, Merial Animal Health Limited) according to the manufacturer’s instructions and quarantined in a slatted pen for a period of 48 h prior to being penned as one group on straw. Information on age and live weight at housing together with the results from the faecal samples collected [25] at housing are summarised in Table 1. Five weeks post-housing and anthelmintic treatment, faecal samples were collected from all animals on 3 consecutive days to determine GIN infection status. Lambs with a positive FEC for “other” trichostrongyles (FEC_OT_), excluding *Strongyloides papillosus*, (two cases: 1 epg and 2 epg) or for *S. papillosus* (FEC_SPAP_; 7 cases: epg ranged from 1 (5 cases) to 50 (2 cases) on any of these days were treated with Ivermectin (Noromectin Drench, Norbrook Laboratories Limited) according to the manufacturer’s instructions and re-sampled to establish the GIN infection status; all were observed to be GIN negative. Approximately 1 week prior to administration of the challenge bolus, lambs were weighed and faecal sampled to confirm the GIN free status.

**Table 1:** **Information (± s.e.) on age, live weight and faecal egg counts for the experimental animals at housing**

**Item**	**Suffolk**	**Texel**	***P*** **value**
No. of lambs	29 (20)^†^	32 (16)^†^	-
Date of birth	28 March	29 March	-
Age at housing (days)	40.2 ± 0.59	39.2 ± 0.56	>0.2
Live weight at housing (kg)	17.7 ± 0.71	17.7 ± 0.68	>0.9
FEC_OT_ (log scale)	4.3 ± 0.25 (41)^‡^	3.6 ± 0.10 (12)^‡^	<0.01
FEC_NEM_ (log scale)	5.8 ± 0.23 (298)^‡^	5.1 ± 0.27 (132)^‡^	<0.05
Live weight 1 week prior to challenge (kg)	46.2 ± 1.51	43.5 ± 1.51	>0.1

^†^No. of lambs that yielded a faecal sample at housing.

^‡^Back-transformed from mean value on log_e_ scale.

### Experimental infection

At approximately 20 weeks of age (~14 weeks post housing), the lambs were randomly assigned within breed, to one of 6 slaughter time points (0, 3, 7, 14, 21 or 35 days post-infection (pi)), with the restriction that members of a twin pair were not assigned to the same time point. The slaughter days were chosen based on the expected developmental stages of *T. circumcincta* in the sheep abomasum; day 3 and day 7 (L4 stage), day 14 (immature adult), days 21 and 35 (mature adult). Animals assigned to the day 0 time point (6 Texel and 4 Suffolk) did not receive an experimental challenge, while all other lambs (5 per breed per time point with an exception of 6 Texels for the day 35 time point) received a single oral dose of 3 × 10^4^ infective *T. circumcincta* larvae (L3). Blood samples were collected into vacutainers with EDTA, lithium-heparin or no anticoagulant, respectively, by jugular vene-puncture immediately prior to slaughter. Plasma was harvested following centrifugation at 2000 *g* for 5 min at 4 °C and stored at −20 °C until use. Blood collected for serum antibody measurement was stored in a refrigerator overnight for clotting. Serum was extracted following centrifugation at 2000 *g* for 5 min and stored at −20 °C until use. Faeces were collected *per rectum* on day 21 and 35 pi and a 3 g aliquot was used to determine the FEC using the modified McMaster method [25]. Animals were slaughtered by electrical stunning followed immediately by exsanguination.

### Worm count

The abomasum was recovered immediately after sacrifice and the contents recovered; the abomasum was then dissected along the greater curvature. A saline-digest technique was used to recover the worms from the abomasal mucosa [26]. Both contents and digest were washed through sieve 1 (75 μm) and sieve 2 (38 μm) followed by preservation in 10% neutral buffered formalin (NBF) [26]. Adult worms were counted from both contents and digests. Extrapolation from two 1% or two 5% aliquots, from sieve 1 and sieve 2 respectively was used for calculating the worm burden.

### Abomasal mast cell and eosinophil counts

A section (approximately 2 cm^2^) of abomasal tissue was taken from the midline fold after the recovery of the abomasal contents. The tissue sample was fixed in 10% NBF and processed in a Sakura Tissue-Tek® VIP processor (Clinical Distributor, Dublin, Ireland). The tissue was sectioned using a Leitz® 1512 Microtome (GMI Inc, mounted on microscopic slides. These sections were stained using GIEMSA (Merck, UK) and examined using a QImaging colour camera (Nikon, Japan) under a 40X objective. The images were visualised using the program Image-Pro. Mast cells and eosinophils were counted in a field area of 0.023 mm^2^. A total of 50 such fields were counted per slide and the mean figure was expressed as total number per 0.023 mm^2^.

### Mucosal antibody recovery

Immediately after slaughter, the surface layer together with the mucus epithelial layer of a portion of abomasal fold tissue (~3 cm^2^), excised from the midline of the dissected abomasum, was removed by scraping with a microscope slide, placed in cryovials and snap frozen in liquid nitrogen followed by storage at −80 °C until use. The samples were prepared for antibody recovery according to the method described previously [27]. Briefly, thawed samples in 3 volumes of phosphate buffered saline (PBS) containing 5 μg/mL of protease inhibitor cocktail (Sigma-Aldrich, St Louis, MO, USA) were homogenised using a Retsch® tissue lyser (Qiagen, Crawley, UK). After centrifugation of the homogenate at 12 000 *g* for 30 min the supernatant was removed and protein concentration determined using the BCA protein assay reagent kit (Pierce, IL, USA). The remaining supernatant was stored at −20 °C.

### IgA ELISA

Antigen from *T. circumcincta* L3 was freshly prepared as previously described [26]. ELISA assays to measure IgA in serum or mucosa were performed as described previously [23]. The wells of 96-well polystyrene ELISA plates (BD FalconTM) were coated with 100 μL of L3 antigen (5 μg/mL) in carbonate buffer at pH 9.6 and stored at 4 °C overnight. The plates were washed 4 times using PBS-T (PBS + 1% Tween). An aliquot of 100 μL of either serum sample (diluted 1:50) or mucosal sample (adjusted to 500 μg/mL) was placed in each well. After plates were washed in PBS 4 times using PBS-T, 100 μL of monoclonal mouse anti-ovine IgA (AbD Serotec, UK), diluted 1:50, was added to each well. Plates were then washed 4 times using PBS-T and 100 μL of a secondary goat anti-mouse horse-radish peroxidase (HRP) conjugate (Dako Diagnostics, Dublin, Ireland) was added to each well and incubated at 37 °C for 30 min. After 4 washes with PBS-T, 100 μL of chromogen, tetramethylbenzidine (TMB) (Dako Diagnostics, Dublin, Ireland) was added to each well and incubated for 15 min at room temperature. The reaction was stopped using 100 μL of 10% 1 M HCl. The AsysTM UVM-240 microplate reader (VWR International, Dublin, Ireland) was used to read the optical density at 450 nm of each plate. Each plate included 3 wells with PBS (TBSA) instead of plasma as a blank, a single serum sample yielding low levels of nematode specific antibodies as a negative control and a single serum sample yielding high levels of nematode specific antibodies as a positive control.

### Pepsinogen and haematology

Plasma pepsinogen concentration was measured as described previously [28]. Whole blood was used for haematology analysis using an ADVIA2120 haematology system (Bayer Healthcare, Leverkusen, Germany) as per manufacturer’s instructions. Neutrophils, eosinophils, monocytes, lymphocytes, basophils, red blood cells (RBC), haemoglobin concentration (Hgb), platelets and mean corpuscular volume (MCV) were measured.

### Statistical analysis

All data were analysed using Proc MIXED (SAS 2003) to fit a model that had effects for breed, time and their interaction. Observations on eosinophils in abomasal sections and on FEC were log transformed prior to analysis to normalise the residuals. Data on differential white cell counts were subjected to the arcsine transformation prior to analysis. Because of the large variation in mean values among subclasses (day or day-by-breed) for many of the variables the heterogeneity of residual variances was tested and accommodated in the mixed model when significant. The results for the F tests of breed and day effects and their interaction are reported but the main interest is the evidence for breed differences in the pattern of change following experimental infection. Thus, the differences among slaughter days were evaluated on a within-breed basis using Dunnett’s test with the lambs slaughtered on day 0 as the control group. Orthogonal polynomials were used to partition the variation between days into single degree of freedom components to describe the pattern of change with time.

## Results

The data on lambs at housing (Table 1) show that there was no difference between the breeds for age or live weight at housing but there was a significant breed effect on FEC_OT_ and FEC_NEM_ (FEC for *Nematodirus battus*). There was no evidence for any association between any of the variables in Table 1 and the slaughter group to which lambs were assigned. All lambs that yielded faecal samples were positive for *N battus*. In the case of “Other trichostrongyles” (excluding S. *papillosus*), positive counts were recorded for 11/16 Texels and 16/20 Suffolks. It is clear from these data that all lambs had experienced some larval challenge while at pasture. There was no significant difference in carcass weights between the Suffolks (21.7 kg) and Texels (21.6 kg).

### Worm burden and FEC

**Figure 1:**
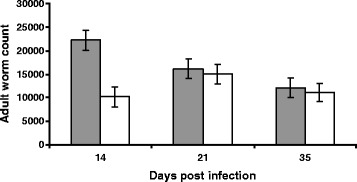
**Adult worm burden.** Adult worm burden (mean ± s.e) in the abomasum of Suffolk (shaded bar) and Texel (clear bar) lambs following infection with 3 × 10^4^ L3 of *T. circumcincta.*

The total number of adult worms recovered on days 14, 21 and 35 pi is presented in Figure 1 for each breed. The mean value was higher for Suffolk lambs on all days and the overall breed effect was significant (*P* < 0.01) but there was a significant breed x day interaction (*P* < 0.05), which reflected the large difference between the breeds at day 14 pi. The FEC values for Texel and Suffolk lambs on day 21 and 35 pi were not significantly different (data not shown).

### Pepsinogen

**Figure 2:**
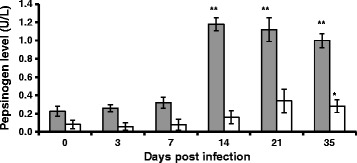
**Serum pepsinogen.** Mean (± s.e.) Serum pepsinogen in Suffolk (shaded bar) and Texel (clear bar) lambs following infection with 3 × 10^4^ L3 of *T. circumcincta*. The asterisks on the bars indicate the significance of the difference from the mean at day 0 for each breed (*P < 0.05, **P < 0.01).

Pepsinogen concentration over the course of the experiment is summarised in Figure 2. Breed and day effects were highly significant (*P* < 0.001) and there was a highly significant (*P* < 0.001) breed-by-day interaction. The interaction reflects the fact that pepsinogen concentration was significantly elevated relative to control in Suffolk lambs on days 14, 21 and 35 whereas there was no significant change in Texel lambs compared with controls until day 35.

### Serum and mucosal IgA

**Figure 3:**
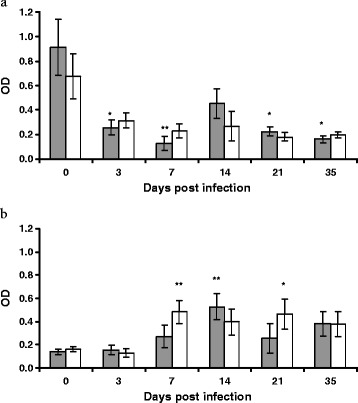
**Serum and mucosal IgA.** Mean (± s.e) OD values for IgA in: **a)** serum and **b)** mucosa of Suffolk (shaded bar) and Texel (clear bar) lambs following infection with 3 × 10^4^ L3 of *T. circumcincta.* The asterisks on the bars indicate the significance of the difference from the mean at day 0 for each breed (*P < 0.05, **P < 0.01).

Levels of serum and mucosal IgA are presented in Figure 3a and 3b respectively. The overall breed effect on serum IgA was not significant but the day effect was significant (*P* < 0.001) with significant quadratic (*P* < 0.05) and cubic components (*P* < 0.01); there was no breed x day interaction. The day effect reflects the fact that levels of serum IgA were highest at day 0 and lower at all subsequent time points in both breeds. Evaluation of the within-breed difference from day 0 showed that serum IgA was significantly lower at all time points except day 14 in Suffolk. While the level of IgA was lower after day 0 in the Texel, this was not statistically significant. The overall breed effect on mucosal IgA was not significant and neither was the breed x day interaction, but the day effect was highly significant (*P* < 0.001) with a significant quadratic component (*P* < 0.05). Evaluation of the within-breed differences from day 0 showed that the mucosal IgA was significantly (*P* < 0.01) elevated by day 7 pi in Texel lambs but no significant elevation was evident in Suffolk lambs until day 14 pi (*P* < 0.01).

### Mast cells and eosinophils in abomasal tissue

**Figure 4:**
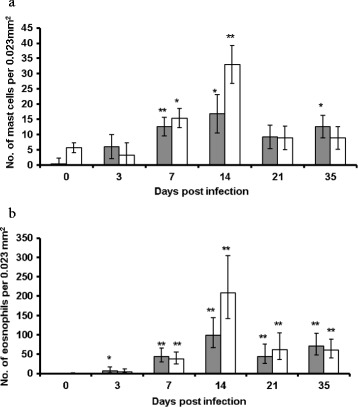
**Abomasal mast cells and eosinophils.** Average number (per 0.023 mm^2^) of **a)** mast cells and **b)** eosinophils in the abomasal tissue of Suffolk (shaded bar) and Texel (clear bar) lambs following infection with 3 × 10^4^ L3 of *T. circumcincta*. The error bars represent the s.e. for mast cells and the 68% confidence interval following back-transformation in the case of eosinophils. The asterisks on the bars indicate the significance of the difference from the mean at day 0 for each breed (*P < 0.05, **P < 0.01).

The average number of mast cells in abomasal tissue from Texel and Suffolk lambs over the course of the experiment is presented in Figure 4a. Mast cells were detected in 5/6 Texel lambs (number per lamb ≤ 13 per 0.023 mm^2^ of abomasal tissue) on day 0 but only a single mast cell was detected in one Suffolk lamb. Mast cells were detected in all animals from day 3 pi onwards and the number peaked at day 14 pi in both breeds; the day effect was significant (*P* < 0.001) and non-linear with an essentially quadratic pattern (*P* < 0.001). Neither breed nor breed-x-day interaction effects were significant. The breed profiles for the number of eosinophils in abomasal tissue over the course of the experiment are shown in Figure 4b. The only occurrence of eosinophils on day 0 was in 3 Texel animals, with low numbers in each case. Neither breed nor breed x day had a significant effect on the number of eosinophils but the day effect was highly significant (*P* < 0.001) with quadratic and cubic components.

### Haematology

**Figure 5:**
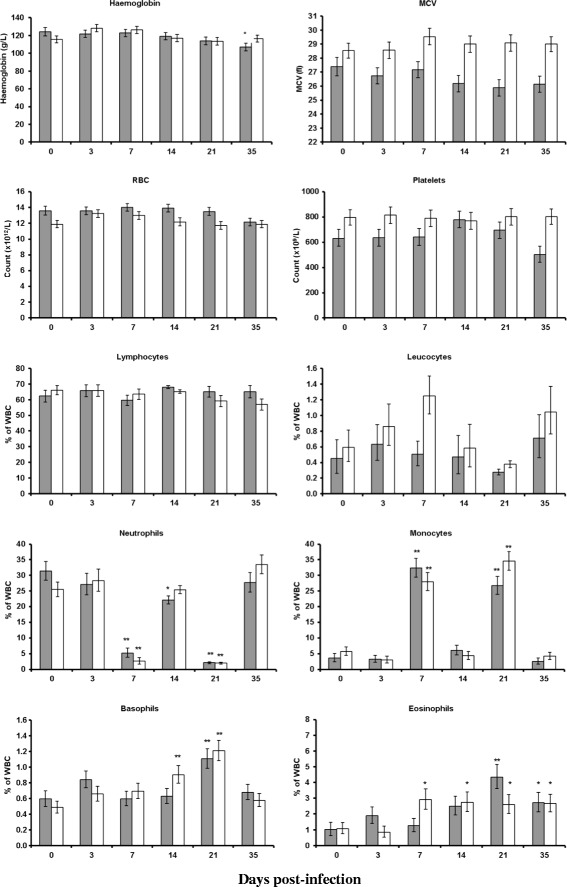
**Hematology variables.** Hematological measurements for Suffolk (shaded bar) and Texel (clear bar) lambs following infection with 3 × 10^4^ L3 of *T. circumcincta.* The error bars for the differential white cell counts represent the 68% confidence interval following back transformation; in the case of the other variables the s.e. is presented. The asterisks on the bars indicate the significance of the difference from the mean at day 0 for each breed (*P < 0.05, **P < 0.01). Percentage means % of a particular cell type out of total white blood cell.

The mean values for Hgb, MCV, RBC, eosinophils, basophils, lymphocytes, leucocytes, neutrophils, monocytes and platelets are presented in Figure 5. There was a significant (*P* < 0.05) day effect for all these variables except lymphocytes, platelets and MCV. The breed-by-day interaction which was significant for eosinophils (*P* < 0.05) probably reflects the earlier emergence of a significant elevation, relative to day 0, in the Texel breed. The breed effect was significant for platelets (Texel > Suffolk; *P* < 0.001), MCV (Texel > Suffolk; *P* < 0.001) and RBC (Suffolk > Texel; *P* < 0.001).

## Discussion

To our knowledge this is the first comparison of the response of Suffolk and Texel lambs to an experimental challenge with *T. circumcincta*. The significant breed difference in worm burden 14 days after artificial infection is consistent with previous observations in this flock on lambs exposed to a natural challenge [6,24]. While the lambs on experiment were not naïve to GIN challenge, the worm burden observed to the experimental challenge would suggest there was not a strong carry over immune effect. The results of this study identified a number of underlying humoral and cellular events that can be argued, contribute to, the observed breed difference in resistance to gastrointestinal nematode infection.

A number of haematological variables, namely RBC, MCV and platelets were found to differ significantly between Texel and Suffolk lambs. The higher number of RBC in Suffolk compared to Texel was consistent with previous findings for these breeds [24]. Recognition for an active role of platelets in both innate and adaptive immune response is increasing [29]. Platelets have been associated with a cytotoxic effect on intravascular parasites [30-33]. The major mechanism is thought to be the parasitic-specific antibody (IgE) binding to platelets followed by platelet activation which results in platelets releasing contents which are toxic for the parasite [34]. While most of the evidence relates to intravascular parasites, there is some evidence for an active role for platelets in patients with extravascular parasites, in particular to *Echinococcus granulosus*. While the precise mechanism remains unclear, variables such as mean platelet volume, (MPV) platelet factor 4 and β-thromboglobulin have been used in the demonstration of platelet activation [35,36]. The significantly greater number of platelets in Texels is consistent with that observed in resistant sheep following *T. circumcincta* infection in a previous study [37] and suggests that platelets may contribute to the resistant phenotype of the Texel.

Neutrophils and monocytes account for approximately 20-30% and 5% of the blood leucocytes in ruminants respectively. Whilst known for their phagocytic activity, they differ in life span and capacity for cytokine production which is associated with the enhancement of the inflammatory response [38]. Circulating monocytes are precursors to specific macrophages and dendritic cell populations at tissue sites of inflammation and infection. Moreover, a recent study concluded that monocytes have potentially both an innate and antigen presentation function in response to different stages of a blood dwelling helminth parasite [39]. In this study, a reduction in the proportion of neutrophils with corresponding increase in the proportion of monocytes was observed at days 7 and 21. The timing of monocytosis observed may reflect the response to different antigenic stimuli (larvae at day 7, adults by day 21).

Peripheral and local eosinophils have been associated with resistance to GIN infection [40,41]. An increase in blood eosinophils in resistant lambs following a mono infection of *T. circumcincta* or *H. contortus* or exposure to a natural challenge has been reported [6,29,41]. Based on these findings an active role for eosinophils in determining the resistance characteristics of the Texel to *T. circumcincta* infection was expected. While there was clear evidence for recruitment of eosinophils into the mucosa in both breeds it is not immediately clear why breed differences for mucosal or circulating eosinophils were absent in the present study.

A predominant characteristic of the cellular response to infection was the increase in abomasal mast cell and eosinophil numbers in both breeds following infection. The fact that mast cells were present in Texels on day 0 and absent in Suffolks could contribute to their more immediate response to GIN challenge. It has been reported previously that mast cells and eosinophils play a vital role in controlling worm burden. It is thought that surface-bound IgE antibodies on the parasite rapidly activate mast cells causing granular release, including eosinophil chemotactic factors, leading to the recruitment of eosinophils and basophils. Subsequent degranulation of mast cells, basophils and eosinophils, results in increased intestinal permeability, greater muscular contractility and subsequent parasite expulsion from the gastrointestinal tract [37,42-45]. An increase in both cell types in gut mucosa during nematode infection in sheep and cattle has been associated with the development of protective immunity against GIN [38]. A recent study in sheep has demonstrated that the number of abomasal mast cells is negatively correlated with the number of adult *T. circumcincta* [46].

Humoral immunity plays a vital role in resistance to GIN infection [24,47,48] and only successful antigen presentation by MHC class II molecules will stimulate an immune response [49]. The lack of significant breed difference in local mast or eosinophil cell numbers may support the proposition that antigen presentation affecting immunoglobulin affinity/avidity may account for the variation in resistance observed between the breeds [24]. In this study, the concentration of circulating IgA declined in both breeds following infection while a concomitant increase in mucosal concentration was observed. The level of circulating IgA in both breeds prior to the experimental challenge may reflect an immune response to the earlier GIN exposure at pasture (turnout after birth to 6 weeks of age) while the experimental challenge led to increased immunoglobulin receptor expression at the mucosal surface to allow translocation of circulating IgA to the abomasal mucosa which was the site of the experimental challenge. Of interest was the observation that Texel sheep had a more rapid mucosal IgA response in comparison to the Suffolk. The within breed analysis showed a significant increase in mucosal IgA concentration by day 7 pi in Texel but not until day 14 pi in Suffolk. This difference in timing may be very important as parasite specific IgA has been previously associated with reduced worm burden, arrested L4 development [47,50] and reduced worm fecundity and length [42]. The evidence suggests that the Suffolk sheep are slower to generate a local humoral immune response which may impair their ability to control worm development. As it has also been suggested that IgE related mechanisms are implicated in the susceptible phenotype of the Suffolk breed [24] further exploration of the humoral response would be of interest.

In this study, pepsinogen concentration was greatly elevated in Suffolks from day 14 pi onwards. In contrast, pepsinogen concentration in Texels was not significantly elevated until day 35 pi and the increase was quite modest when compared with that observed in Suffolk lambs. These data support previous findings which suggested that GIN infected Suffolks have higher concentrations of circulating pepsinogen than co-grazed Texels [24]. Pepsinogen concentrations greater than 1 U/L are considered clinically significant [51,52]; the concentration of pepsinogen recorded for Suffolks suggests that these animals suffered much greater abomasal damage than Texels, which may have long-term negative consequences for the Suffolk in terms of ability to manage subsequent challenges and to maintain growth. Support for this hypothesis can be taken from results of a previous study on the effect of contrasting levels of parasite challenge on performance; it was found that the Suffolk exhibited a greater growth penalty compared to Texel in the presence of a high level of parasite challenge [53].

In summary, while overall recruitment patterns of mucosal mast cells and eosinophils were similar between breeds, the evidence showed that exposure of infective larvae to mucosal IgA occurred earlier in the Texel breed. It is suggested that this may play a role in the more effective control of worm burden in the Texel. The Suffolk breed had significantly higher worm burden at day 14 and elevated pepsinogen concentration from day 14 onwards. It is speculated that the greater damage to the mucosa observed in the Suffolk would render Suffolk lambs more susceptible to the on-going larval challenge that would occur under normal grazing conditions and so contribute to the susceptible phenotype of the Suffolk breed.
